# A Grub to Catch Gudgeons

**Published:** 1886-04

**Authors:** 


### '' A GRUB TO CATCH GUDGEONS.''

It makes us smile to see with what avidity certain of our contemporaries have swallowed a bait temptingly held out by a certain concern that deals in optical instruments. This concern sent its catalogue and a letter to a number of medical journals. We received them. The letter said: ''As we may, at some future time, *no doubt*, use your advertising pages [etc.] please give us an editorial notice, calling especial attention to our facilities for fitting glasses by physicians' prescriptions,'' etc. We see several of our contemporaries have, accordingly, puffed the concern, on such a vague promise. We replied: '' When that future time *shall have arrived*, and you *do* use our advertising pages (and *pay for them*), we may then ' give you an editorial notice;'' not before. We have neither the inclination, leisure nor space to puff anybody's enterprises, except those of our patrons. Yours truly.''

